# The subcellular distribution of myeloid-related protein 8 (MRP8) and MRP14 in human neutrophils

**DOI:** 10.1186/1479-5876-3-36

**Published:** 2005-09-28

**Authors:** David F Stroncek, Raji A Shankar, Keith M Skubitz

**Affiliations:** 1 Department of Transfusion Medicine, Warren G. Magnuson Clinical Center, National Institutes of Health, Bethesda, Maryland, USA; 2 3M Corporation, St. Paul, Minnesota, USA; 3 The Department of Medicine, The University of Minnesota Medical School and Masonic Cancer Center, Minneapolis, Minnesota, USA

**Keywords:** Myeloid-related protein 8, MRP8, myeloid-related protein 14, MRP14, neutrophils, macrophages, calprotectin

## Abstract

**Background:**

Myeloid-related protein 8 (MRP8) and MRP14 are S100 family calcium binding proteins that form a heterodimer known as calprotectin or MRP8/14 that is present in the cytosol of neutrophils and monocytes. MRP8/14 becomes associated with endothelium at sites of monocyte and neutrophil adhesion and transmigration and induces a thrombogenic and inflammatory response by increasing the endothelial transcription of proinflamatory chemokines and adhesion molecules. The distribution of MRP8/MRP14 among neutrophil granules and plasma membranes is unclear and was investigated to better understand the role of this molecule in acute inflammation.

**Study design:**

Three monoclonal antibodies specific for MRP8 and MRP14 were characterized and used in immunoblotting assays of neutrophil whole cell extracts, and isolated plasma membranes, primary granules, secondary granules and cytosol.

**Results:**

MRP8 and MRP14 were detected in neutrophil cytosol, plasma membrane, primary granule and secondary granule fractions. MRP8/14 demonstrated a calcium-dependent adherence to plasma membranes and primary granules and could be removed by washing with EGTA in a high ionic strength buffer. In contrast, MRP8/14 was found within the contents of the secondary granules. Activated neutrophils released secondary granules and MRP8/14.

**Conclusion:**

MRP8/14 is located in neutrophil cytosol and secondary granule fractions and is loosely associated with plasma membranes. MRP8/14 released with secondary granules by activated neutrophils likely binds to endothelium and plays an important role in acute inflammation.

## Introduction

Calprotectin is a heterodimer of two calcium-binding proteins that belong to the S100 family: myeloid-related protein 8 (MRP8) and MRP14. MRP8 (10.8 kDa) is also known as p8, L1 light chain, calgranulin A, and cystic fibrosis antigen (CFA). MPR14 (13.2 kDa) is also referred to as p14, L1 heavy chain, and calgranulin B [1-7]. MRP8 and MRP14 bind both calcium and zinc. Calprotectin is also known as MRP8/14.

Neutrophils are the major producers of calprotectin, but monocytes and some macrophages express MRP8/14. Macrophages found at the sites of acute infection express MRP8/14, but resident tissue macrophages and those found at the sites of chronic inflammation do not [7]. MRP8/14 is found in the cytosol of neutrophils and macrophages[8,9]. It is the most abundant protein in neutrophil cytosol making up 30% to 60% of all cytosolic proteins[8], but it is much less abundant in monocytes and comprises about 1% of all monocyte cytosol protein [10].

MRP8/14 plays an important role in leukocyte interactions with endothelium. It is not expressed by endothelium, however, examination of tissue sections revealed that MRP8/14 becomes associated with endothelium at sites where monocytes and neutrophils pass through the endothelium [7]. *In vitro *studies have found that when activated monocytes come in contact with extracellular matrix proteins or inflamed endothelium intracellular calcium levels increase, protein kinase C is activated, and MRP8/14 is released [11,12]. Once MRP8/14 is released it binds to the endothelium. Two mechanisms of binding have been proposed. One group has shown that MRP14 binds to endothelial cell heparan sulfate proteoglycans[13] and another that MRP8/MRP14 binds to carboxylated N-glycans expressed by activated endothelial cells [14].

Once associated with the endothelium, MRP8/14 has important proimflamatory effects. The binding of MRP8/14 to endothelium induces a thrombogenic and inflammatory response by increasing the transcription of proimflamatory chemokines and adhesion molecules and by decreasing the expression of cell junction proteins and molecules involved in cell monolayer integrity [15].

Elevated levels of MRP8/14 have been found in many sites of inflammation and in the extracellular fluid of patients with many types of inflammatory conditions. The concentration of MPR8/14 in the blood is increased in patients with rheumatoid arthritis, Chron's disease, colorectal cancer, cystic fibrosis, multiple scerlosis, and HIV infections [1,2,4,6,16]. Extracellular MRP8/14 has antimicrobial, antigrowth and apoptotic effects. It suppresses the growth of some fungi and bacteria [1,2]. It also suppresses the proliferation of several different types of cells including: macrophages, lymphocytes, hematopoietic progentitors, and tumor cell lines. MRP8/14 can also induce apoptosis of some tumor cell lines [1,2].

The primary source of MRP8/14 in tissues and body fluids has been suggested to be the cytosol of dead or lysed neutrophils [2]. However, the source of neutrophil MRP8/MRP14 that becomes associated with endothelium is not certain. While MRP8/14 is the most abundant neutrophil cytosol protein, investigations of the distribution of MRP8/14 among neutrophil granules and plasma membranes have yielded conflicting results. Some studies have reported that MRP8/14 is present in neutrophil cytosol and plasma membranes, but the primary or secondary granules were not analyzed [3,17]. However, another study did not detect MRP8/14 in either the plasma membranes or granules [10].

This study analyzed the neutrophil subcellular distribution of MRP8 and MRP14 to better understand changes in expression of MRP8/14 by activated neutrophils and the role of neutrophil MRP8/14 in acute inflammation. The expression of MRP8/14 on neutrophil plasma membranes, primary granules, secondary granules and cytosol was analyzed.

## Materials and methods

### Isolation of Neutrophils

Neutrophils were isolated by dextran sedimentation and centrifugation over Ficoll-Hypaque using a modification of the method of Boyum [18,19] and suspended in phosphate-buffered saline (PBS) or Hank's balanced salt solution (HBSS; GIBCO, Grand Island, NY).

### Production of Monoclonal Antibodies

The monoclonal antibodies (MoAbs) were produced using BALB/c mice as previously described [20]. MoAbs AHN-17, AHN-17.1, and 15H9 were produced using human neutrophils as the immunogen, and AHN-17.1 using human eosinophils obtained from a patient with hypereosinophilic syndrome.

### Characterization of Monoclonal Antibodies

All 3 MoAbs were IgG1. The MoAbs were tested by immunostaining [21] against neutrophil slide preparations, prepared by cytocentrifugation and all three reacted with neutrophils. AHN-17 and AHN-17.1 were used as ascites and 15H9 as isolated immunoglobulin.

### Immunoblotting of Neutrophil Proteins

Neutrophils (1 × 10^8 ^per mL of PBS) were treated with 5 mmol/L diisopropylfluorophosphate (DIFP) (Sigma Chemical Company, St. Louis, MO) for 10 minutes at 4°C and were washed twice with PBS. The cells were resuspended at a concentration of 5 × 10^7 ^cells/mL in a buffer containing 5 mM Tris (Sigma), 400 mM KCl, 1 mM EDTA (Sigma), 1% Triton-X-100, and 2 mM (PMSF) (Sigma), pH 8.2 (Extraction Buffer) [20]. The solution was then frozen, thawed, and centrifuged at 10,000 g, for 10 minutes at 4°C. The cell extract was then analyzed by sodium dodecyl sulfate-polyacrylamide gel electrophoresis (SDS-PAGE), transferred to nitrocellulose, and immunoblotted as previously described [22]. SDS-PAGE was performed in a 13% gel and the cell extracts were analyzed under both reducing and non-reducing conditions. MoAb 15H9 was used at a final concentration of 10 μg/mL and the ascites containing AHN-17 and AHN-17.1 at a dilution of 1:300.

### Subcellular Fractionation of Neutrophils

Subcellular fractionation of neutrophils was performed using a modification of the method described by Borregaard et al [22,23]. Briefly, neutrophils were resuspended in 10 mL ice cold Relaxation Buffer [100 mM KCI, 3 mM NaCl, 1 mM adenosine triphosphate (Na)_2 _(Sigma), 3.5 mM MgCl_2_, and 10 mM piperazine-N, N-bis (2-ethanesulfonic acid) (PIPES) (Sigma), pH 7.3]. This solution was then equilibriated for 20 minutes at 4°C with nitrogen at 350 PSI with constant slow stirring in a cell disruption bomb (Parr Instrument, Moline, IL Model no 4639). The suspension was then collected drop wise into 2.2 mL of 12.5 mM EDTA (Sigma), pH 7.4 in Relaxation Buffer. Nuclei and unbroken cells were removed by centrifugation at 500 g for 20 minutes at 4°C. Percoll (Pharmacia Fine Chemicals, Piscataway, NJ) was adjusted to a density of 1.120 and 1.050 g/mL with relaxation buffer 10 times concentrated. The 1.050 density Percol was laid over the 1.120 density Percol, and the supernate was loaded onto the Percol gradients, which had been precooled at 4°C. The pelleted membranes and granules were resuspend in Relaxation Buffer and centrifuged at 100,000 g for 90 minutes at 4°C. The membranes and granules were washed again with relaxation buffer and analyzed by immunoblotting. The plasma membrane, primary granule, secondary granule, and cytosol fractions were analyzed by immunoblotting with AHN-1.1 (CD15) [20], AHN-10 (anti-elastase) [24], AHN-9 (anti-lactoferrin) [21], and AHN-11 (anti-cathepsin G) [21] to confirm that adequate separation had occurred.

In other studies the plasma membranes, primary, and secondary granules were centrifuged at 100,000 g for 90 minutes at 4°C and resuspended in 1 M NaCl and 10 mM EGTA (Sigma), pH 7.4 in Relaxation Buffer. The wash was repeated and then the plasma membranes, primary granules and secondary granules were disrupted by freeze thawing twice and sonicating (Microultrasonic Cell Disrupter, model KT40 Kontz) for 1 minute on ice. The membranes and soluble granule contents were separated by centrifugation at 120,000 g for 90 minutes at 4°C. Protein concentrations of the fractions were determined by bicinchoninic acid (BCA) protein assay C (Pierce Chemical Company, Rockford, IL).

### Stimulation of Neutrophils

Neutrophils at a concentration of 10^7^/mL in HBSS were incubated at 37°C for 15 minutes alone or with phorbol myristate acetate (PMA) (100 ng/mL) (Sigma) or N-formal-Met-Leu-Phe (FMLP) (10^-6^M) (Sigma). The solution was then centrifuged at 500 g for 10 minutes and the supernatant was analyzed by immunoblotting.

### Isolation of Proteins

MoAbs AHN-17 and 15H9 were immobilized on a protein A column and crosslinked with dimethyl pimelimidate according to the manufacturer instructions (lmmuno Pure IgG Orientation kit (Pierce). Neutrophils or subcellular fractions were suspended at a concentration of 5 × 10^7^/mL in Extraction Buffer. The solution was then frozen, thawed, and centrifuged at 10,000 g for 10 minutes at 4°C. The neutrophil extact was mixed 1:1 with 10 mM Tris, ph 7.5, and applied to the immunoaffinity column. After the column was washed with 150 mM NaCl, 0.2% Triton X-100, 10 mM Tris, pH 7.5 to remove unbound protein. Antigens were eluted with 0.1 M glycine, pH 2.8, containing 0.2% Triton X-100 and immediately neutralized with 2 M Tris-HCI, pH 7.6.

The 10 kDa and 14 kDa proteins isolated with MoAb AHN-17 were analyzed by SDS-PAGE and coomassie blue staining or immunoblotting. The 10 and 14 kDa proteins isolated with MoAb 15H9 were separated by reverse phase high pressure liquid chromatography (HPLC) using C-4 column (Vydac, Hesperia, CA) which had been pre-equilibrated with three column volumes of 0.1% trifluoroacetic acid. The bound proteins were eluted from the column by application of 0–60% gradient of acetonitrite over 50 minutes. Aliquots (1 mL) were collected and analyzed by SDS-PAGE and silver staining or immunoblotting with MoAb 15H9. The N- terminal amino acid sequence of the 10 and 14 kDa proteins were analyzed using an automated amino acid sequencer (ABl 470/477, Applied Biosystem Inc, Foster City, CA).

### Tryptic Digestion of the 14 kDa Protein

The isolated 14 kDa protein was dried using vacuum centrifugation. The dried proteins were resuspended in 50 μL of a solution containing 8 M urea and 0.4 M ammonium bicarbonate and following the addition of 10 μL 45 mM dithiothreitol (Sigma) was incubated for 30 minutes at 50°C. After the solution cooled to 20°C, 10 μL of 100 mM iodacetamide (Sigma) was added and the solution was incubated for 30 minutes in the dark at 20°C. Next, trypsin (Worthington Biochemical Corp, Freehold, NJ) in 120 μL of water at 1:25 (w/w) ratio of enzyme to protein (determined by BCA) was added and the solution was incubated at 37°C for 24 hours. The resulting tryptic fragments were separated by high pressure liquid chromatography (HPLC) (C-18 Column, Vydac, Hesperia, CA). The N-terminal amino acid sequence of the tryptic fragments was analyzed using an automated amino acid sequence (Applied Biosystems).

## Results

### Characterization of MoAbs AHN-17, AHN-17.1, and15H9 as Specific for MRP8 and MRP14

All three MoAbs were tested in an immunoblotting assay against neutrophils analyzed by SDS-PAGE under non-reducing conditions. Multiple proteins of various apparent molecular weights were detected by AHN-17, AHN-17.1, and 15H9 and the pattern of proteins detected was similar for all three MoAbs (data not shown). AHN-17 and AHN-17.1 did not react by immunoblotting with proteins from extracts analyzed by SDS-PAGE under reducing conditions, but 15H9 reacted with two proteins of 10 kDa and 14 kDa (data not shown). These results suggest that the three antibodies react with same proteins, but the eptiopes recognized by AHN-17 and AHN-17.1 are confirmation dependent.

**Figure 1:**
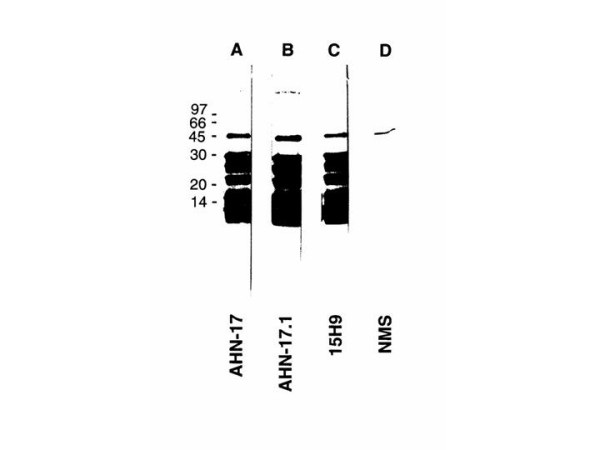
Immunoblotting of proteins purified by affinity chromatography using monoclonal antibody AHN-17. Neutrophil proteins recognized by AHN-17 were isolated by immunoaffinity chromatography, analyzed by SDS-PAGE in a 13% gel under non-reducing conditions, transferred to nitrocellulose, and immunoblotted with AHN-17 (lane A), AHN-17.1 (lane B), 15H9 (lane C), and normal mouse serum (NMS) (lane D). Proteins used as molecular weight standards were: rabbit muscle phosphorylase B, 97,000; Bovine serum albumin 66,000; ovalbumin 45,000; glyceraldehyde 3 phosphate dehydrogenase, 36,000; bovine erythrocyte carbonic anhydrase 30,000; soybean trypsin inhibitor 20,000; and bovine lactalbumin 14,000.

To determine if all three MoAbs reacted with the same proteins, the antigens recognized by AHN-17 were isolated by affinity chromatography with AHN-17 and were analyzed by immunoblotting with AHN-17, AHN-17.1, and 15H9. When the purified proteins were analyzed by SDS-PAGE under non-reducing conditions and coomassie blue staining, several bands were detected but when analyzed under reducing conditions only a 10 kDa and 14 kDa protein were detected (data not shown). When the isolated proteins were analyzed by SDS-PAGE under nonreducing conditions and immunoblotting, AHN-17 (Figure 1, lane A), AHN-17.1 (lane B) and 15H9 (lane C) reacted with several bands and the proteins identified by all three antibodies were similar. Immunoblotting with normal mouse serum (NMS) did not detect these proteins (lane D). These results indicate that all three antibodies recognized the same proteins.

**Figure 2:**
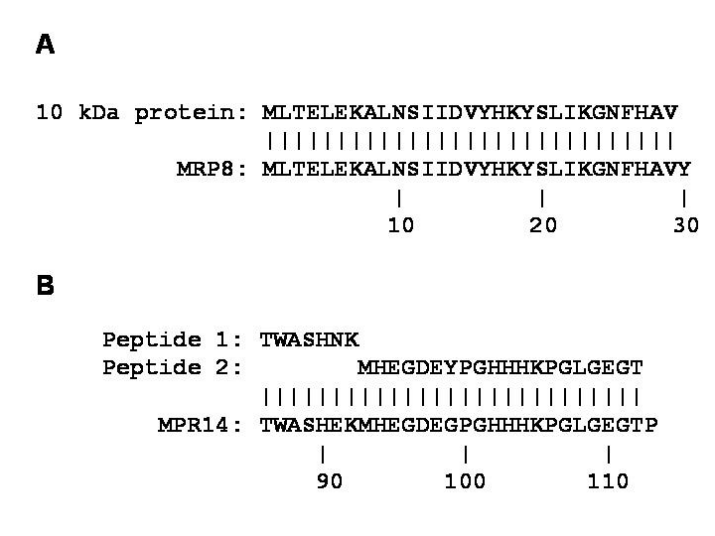
Comparison of the N-terminal amino acid sequences of the 10 kDa protein and MRP8 (panel A) and the amino acid sequences of the two tryptic peptides from the 14 kDa protein with MRP14 (panel B).

The 10 and 14 kDa proteins purified by affinity chromatography with MoAb AHN-17 were separated by HPLC, and the N-terminal amino acid sequence of the 10 kDa protein was analyzed. A sequence of 29 amino acids was identified which was identical to that of MRP8 (Figure 2). The N-terminus of the 14 kDa protein was blocked, however, the amino acid sequences of two tryptic peptides were determined and found to be identical to MRP14 at 25 of 27 amino acids (Figure 2).

### Subcellular Distribution of MRP8 and MRP14 within Neutrophil Granules

**Figure 3:**
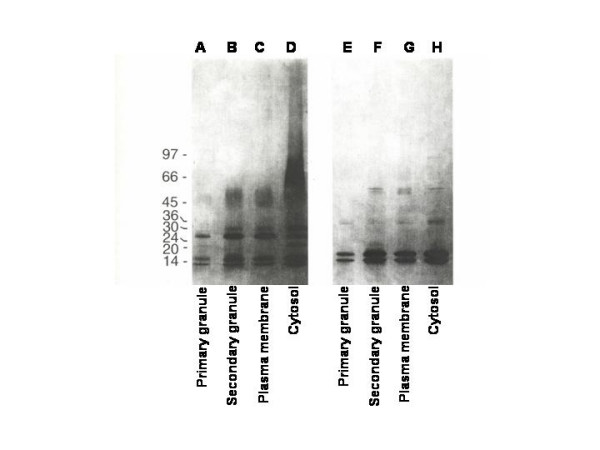
Subcellular distribution of proteins recognized by antibody AHN-17. Primary granules, secondary granules, plasma membranes, and cytosol were isolated from the neutrophils by nitrogen cavitation and differential centrifugation and were suspended in extraction buffer. The antigens recognized by antibody AHN-17 were isolated from each fraction by affinity chromatography with ANH-17 and were analyzed by SDS-PAGE in a 10% gel under non-reducing (lanes A-D) and reducing conditions (lanes E-H). The amount of antigen applied to each lane was adjusted to represent the same percentage of starting material. Primary granules are shown in lanes A and E, secondary granules in lanes B and F, plasma membranes in lanes C and G, and cytosol in lanes D and H. Gels were stained with coomassie blue.

Nitrogen cavitation and density gradient separation was used to isolate neutrophil cytosol, plasma membranes, primary granules, and secondary granules. The secondary granule fraction also contains a distinct granule population termed tertiary or gelatinase granules [25]. The proteins recognized by AHN-17 were isolated from each of these fractions by affinity chromatography and analyzed by SDS-PAGE under reducing and non-reducing conditions. When the proteins were analyzed by SDS-PAGE under non-reducing conditions, molecules of several electrophoretic mobilities were detected (Figure 3, lanes A-D). However, only two proteins of 10 and 14 kDa were detected when the analysis was preformed under reducing conditions (Figure 3, lanes E-H). These studies show that MRP8 and MRP14 are located in the neutrophil cytosol, plasma membrane, and secondary granule, and to a lesser degree primary granule fractions.

**Figure 4:**
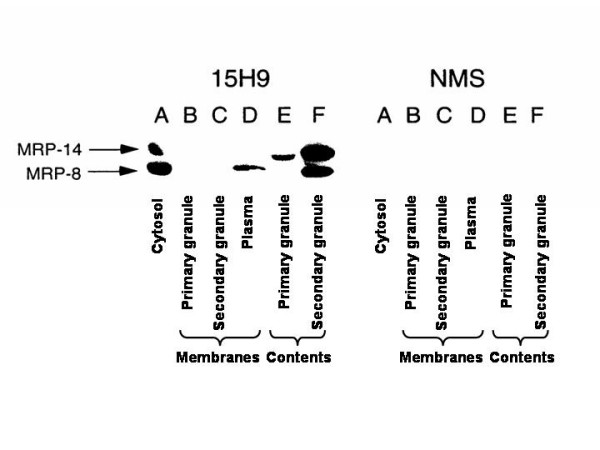
Analysis of neutrophil cytosol, plasma membranes, primary granules, and secondary granules by immunoblotting with monoclonal antibody 15H9. Neutrophil plasma membranes, cytosol, primary granules and secondary granules were prepared by nitrogen cavitation and differential centrifugation. Primary and secondary granules were disrupted and the membrane and contents were separated as described in the text. Cytosol (lane A), primary granule membranes (lane B), secondary granule membranes (lane C), plasma membranes (lane D), primary granules contents (lane E), and secondary granule contents (lane F) were analyzed by SDS-PAGE in a 13% gel under reducing conditions and were immunoblotted with antibody 15H9 (left panel) or NMS (right panel). Five micrograms of protein was added to each lane except for lane D in which 2 micrograms of protein was analyzed.

To further determine the location of the proteins in the cellular granules, primary and secondary granules were washed and disrupted by freeze-thawing and sonication and the membranes were separated from the granule contents by centrifugation as described in the methods section. Equal amounts of protein from the cytosol, plasma membranes, primary granule membranes, secondary granule membranes, and the soluble contents of the primary and secondary granules were analyzed by immunoblotting with 15H9 (Figure 4). Both MRP8 and MRP14 were detected by 15H9 in the cytosol (Figure 4, first panel, lane A) and the soluble portion of the primary and secondary granules (Figure 4, first panel, lane E and F). MRP8 was also detected on the plasma membrane fractions (Figure 4, first panel, lane D). The largest quantities of the MRP8 and MRP14 were present in cytosol and in the soluble portion of the secondary granules. In contrast, NMS did not react with any proteins of similar electrophoretic mobility (Figure 4, second panel lanes A through F).

These studies were repeated but the plasma membranes, primary granules and secondary granules were washed two additional times with a high ionic strength buffer containing EGTA to disrupt loose associations of MRP8 and MRP14 with plasma membranes and granules. After the primary and secondary granules were disrupted by freeze thawing and sonication, the plasma membranes, secondary granules, and soluble contents of the granules were analyzed by immunoblotting. MRP8 and MRP14 were present only in the soluble portion of the secondary granules, but not the plasma membranes or primary granules (data not shown).

The neutrophil plasma membrane, cytosol, and granule fractions were also immunoblotted with AHN-10 (anti-elastase), AHN-9 (anti-lactoferrin), AHN-11 (anti-cathepsin G), and AHN-1.1 (CD15) to confirm adequate fractionation. As expected, elastase and cathepsin G were found in the primary granule contents, and lactoferrin in the secondary granule contents. The CD15 antibody reacted with plasma membranes and secondary granule membranes as expected (data not shown).

To examine the ability of neutrophils to release MRP8/14 when activated neutrophils were stimulated with PMA or FMLP to release secondary granules and tertiary granules, the cell suspension was centrifuged and the supernatant was analyzed by immunoblotting with MoAb 15H9. A small amount of MRP8 and MRP14 was detected in the supernatant of cell incubated in HBSS, however, the quantities of MRP8 and MRP14 in the supernatant was increased significantly after stimulation with PMA and FMLP (data not shown).

## Discussion

The expression of MRP8 and MRP14 in neutrophils was analyzed using MoAbs AHN-17, AHN-17.1, and 15H9. Antibody specificity was confirmed by amino acid sequencing; the N-terminal amino acid sequence of the 10 kDa protein recognized by the antibodies was found to be identical to MRP8. The N-terminus of the 14 kDa protein recognized by the antibodies was blocked, but amino acid sequencing of tryptic peptides showed that the protein was MRP14. MRP8 and MRP14 have a similar structure and other antibodies have been found to react with both proteins [8,9]. MRP8 and MRP14 are encoded by two separate genes located on chromosome 1q12-q21[26].

This study confirmed that MRP8/14 are in neutrophil cytosol and found that MRP8 and MRP14 are also in the secondary granule fraction with a much smaller amount in the primary granule fraction and on the plasma membrane. The secondary granule fraction also contains the so-called tertiary granules or gelatinase granules [25]. The association of MRP8/14 with neutrophil plasma membranes and primary granules was similar to the association of MRP8/14 with macrophage plasma membranes [2,27]. Thus while in macrophages MRP8/14 is found primarily in cytosol, its distribution of depends on the activation state of the macrophages. In resting macrophages MPR8/14 is in the cytosol, but when macrophages are stimulated, it translocates to the cell membrane and cytoskeleton. The association of MRP8/14 with macrophage membranes is calcium dependent [27]. We found that MRP8 and MRP14 had a loose calcium-dependent adherence to neutrophil primary granules and plasma membranes and they were removed from these membranes by washing with EGTA in a high ionic strength buffer. Lemarchand and coworkers also found that MRP8/14 was associated with neutrophil plasma membranes via a calcium-dependent mechanism [28].

The association for MRP8/14 with secondary granules was different than its association with plasma membranes and primary granules; MRP8/14 was within the contents of the secondary granules. It could not be removed from the secondary granules by EGTA washes and when secondary granule membranes were separated from their soluble contents, almost all of the MRP8/14 was found to be present in the soluble contents of the secondary granules.

We found that MRP8/14 is released within seconds from secondary and or tertiary granules by stimulated neutrophils. MRP8/14 was detected in the supernatant of cells incubated with buffer possibly due to the loss of MRP8/14 associated with plasma membranes. However, following the incubation of cells with stimulants known to cause the release of secondary granules and tertiary granules, MRP8/14 was released from neutrophils. The subcellular fractionation technique used in this study separated neutrophils into cytosol, primary granule, secondary granule and plasma membrane fractions. The secondary granule fraction also likely contained tertiary granules [25,29,30], and MRP8 and MRP14 may also be present in tertiary granules.

It is not certain why other studies did not detect MRP8 and MRP14 in neutrophil granules [3,10,17]. The structure of MRP8 and MRP14 in the cytosol may differ from the structure of granule MRP8 and MRP14, and it is possible that some antibodies may recognize the cytosol proteins but not the granule proteins. Alternatively, the state of neutrophil activation may have differed between the studies. While MRP8/14 is associated with plasma membranes, they are not integral membrane proteins. Neither has a membrane-anchor sequence [4]. When neutrophils are activated, the quantities of both MRP8 and MRP14 associated with plasma membranes increases [28].

The presence of MRP8/14 in neutrophil secondary granules suggests that neutophils contribute to the deposition of MRP8/MRP14 on endothelium. Since adherent neutrophils release secondary granules, it is likely that at sites of acute inflammation when neutrophils bind to endothelial cells they degranulate and release MRP8/14 which stimulates endothelium and induces thrombotic and proimflamatory changes. This suggests that MRP8/14 has an important role in neutrophil mediated inflammation.

In summary, MRP8 and MRP14 are located not only in neutrophil cytosol but also in secondary and/or tertiary granules. The release of secondary granules and MPR8/14 by activated neutrophils suggests that MRP8/14 plays an important role in neutrophil induced inflammation.
